# Overexpression of FOXM1 predicts poor prognosis and promotes cancer cell proliferation, migration and invasion in epithelial ovarian cancer

**DOI:** 10.1186/1479-5876-12-134

**Published:** 2014-05-20

**Authors:** Ning Wen, Yu Wang, Li Wen, Shu-Hua Zhao, Zhen-Hua Ai, Yi Wang, Bo Wu, Huai-Xiu Lu, Hong Yang, Wen-Chao Liu, Yu Li

**Affiliations:** 1 Department of Stomatology, The General Hospital of Chinese PLA, Beijing 100853, China; 2 Department of Oncology, State Key Discipline of Cell Biology, Xijing Hospital, The Fourth Military Medical University, Xi’an 710032, China; 3 Department of Obstetrics and Gynecology, Xijing Hospital, The Fourth Military Medical University, Xi’an 710032, China; 4 Cell Engineering Research Centre and Department of Cell Biology, State Key Laboratory of Cancer Biology, The Fourth Military Medical University, Xi’an 710032, China; 5 Department of Stomatology, Navy General Hospital, Beijing 100048, China

**Keywords:** Ovarian cancer, FOXM1, Prognosis, Proliferation, Migration, Invasion

## Abstract

**Background:**

The Forkhead box M1 (FOXM1), an important regulator of cell differentiation and proliferation, is overexpressed in a number of aggressive human carcinomas. The purpose of this study was to examine the expression levels of FOXM1 in epithelial ovarian cancer (EOC), to identify the relationship between FOXM1 expression and patient survival, and to investigate the role of FOXM1 in human ovarian cancer development.

**Methods:**

Immunohistochemical analysis for FOXM1 was performed in a total of 158 ovarian tissue specimens, all with linked clinical outcome data. Kaplan–Meier method and Cox proportional hazards analysis were used to relate FOXM1 expression to clinicopathological variables and to progression-free survival (PFS) and overall survival (OS). *In vitro* studies were performed to determine the function of FOXM1 in cell proliferation, migration and invasion in EOC cells using pcDNA3.1-FOXM1 and FOXM1 shRNA.

**Results:**

Elevated FOXM1 levels were associated with lymph node metastasis (*P* = 0.009), but not with age, FIGO stage, histological grade and histological type. Patients with high expression of FOXM1 had poorer PFS (*P* = 0.0001) and OS (*P* < 0.0001) than patients with low expression of FOXM1. Furthermore, multivariate analyses indicated that FOXM1 positivity was an independent prognostic factor for PFS (*P* = 0.046) and OS (*P* = 0.022), respectively. Overexpression of FOXM1 increased expression and activity of matrix metalloproteinase-2 (MMP-2), MMP-9 and vascular endothelial growth factor-A (VEGF-A), and cancer cell proliferation, migration and invasion of HO-8910 cells, whereas knockdown of FOXM1 reduced expression and activity of MMP-2, MMP-9 and VEGF-A, and cancer cell proliferation, migration and invasion of HO-8910 PM cells.

**Conclusions:**

Our results suggest that FOXM1 expression is likely to play important roles in EOC development and progression. FOXM1 expression is a potential prognostic factor for PFS and OS, and it could be a novel treatment target in EOC patients.

## Background

Ovarian cancer is the leading cause of death among gynecologic malignancy in the world, with increasing incidence recently in China [1]. Most patients are diagnosed with late-stage disease due to the silent early stage and easy metastasis [2]. Epithelial ovarian cancer (EOC) account for nearly 70% of all ovarian malignant diseases. Despite improvements in surgical techniques and the advent of more targeted therapeutic agents, therapeutic failure and disease progression are still quite frequent [3]. Therefore, there is an urgent requirement to identify extra prognostic indicators and to improve on current understanding of the molecular mechanisms underlying EOC. It would be very helpful for patients with advanced disease.

Forkhead box protein M1 (FOXM1), also known as HFH-11, MPP-2, WIN, and Trident, is a typical transcription factor that belongs to the Forkhead Box family, which is evolutionarily conserved and is defined by having a common DNA-binding domain called Forkhead or winged-helix domain [4,5]. FOXM1 is well-known for its critical role in cell cycle progression by regulating the transition from G1 to S phase and G2 to M phase progression, as well as to mitosis [5,6]. Gene expression profiling revealed that elevated expression of FOXM1 was observed in a multitude of malignancies [7]. It has been reported that FOXM1 promotes tumor progression in malignancies [5,8-11]. Furthermore, overexpression of FOXM1 correlated with disease progression and poor prognosis and could serve as an independent predictor of poor survival in various human malignancies [12-16]. However, the clinical relevance of FOXM1 expression has not been investigated in patients with EOC.

In this study, we used an immunohistochemical method to determine the expression of FOXM1 in EOC specimens from 158 patients. The aim of this study was to determine the expression level of FOXM1 in EOC and to examine its association with clinicopathologic variables as well as to assess the utility as prognostic indicator. We further probed the biological features of FOXM1 by assaying cell proliferation, migration, invasion and potential mechanism underlying this. In this report, we showed that up-regulation of FOXM1 increased MMP-2, MMP-9 and VEGF-A expression whereas knockdown of FOXM1 by FOXM1 shRNA decreased MMP-2, MMP-9 and VEGF-A expression. In addition, the results supported a mechanism for the FOXM1-induced cell migration and invasion that involved induction of MMP-2, MMP-9 and VEGF-A expression. The results presented here help to evaluate the suitability of FOXM1 as prognostic marker and therapeutic target in EOC.

## Methods

### Patients and tissue samples

The study has been performed with the approval of the Fourth Military Medical University ethics committee. Informed consent was obtained from each patient before sample collection. For real-time PCR and western blot analysis, we collected data on 68 patients, including epithelial ovarian cancer (46 cases) and normal ovary (22 cases), treated at Xijing Hospital between November 2009 and June 2011. For immunohistochemical analysis, a total of 158 pathological specimens of EOC were obtained from the pathological archives at Xijing Hospital between May 2004 and July 2006. None of patients had received preoperative chemotherapy. The median age of patients at the time of surgery was 53 years (range, 26–79 years). Histological grades were assigned according to the International Federation of Gynecology and Obstetrics (FIGO) classification. The follow-up interval was calculated from the date of surgery to the date of death or last clinical evaluation. Tumour progression was defined based on clinical, radiological or histological diagnosis.

### Immunohistochemistry

Immunohistochemical staining was performed using the streptavidin-peroxidase conjugate method. Surgical specimens were fixed in 10% formalin and embedded in paraffin. Briefly, after 4 μm-thick sections were deparaffinized in dimethylbenzene and rehydrated in an alcohol series, antigens were retrieved by boiling in a citrate buffer (0.01 M, pH = 6.0). Endogenous peroxidase activity was blocked with 0.3% hydrogen peroxide in methanol, and nonspecific immunoglobulin binding was blocked by incubation with 10% normal goat serum for 15 min. After rinsing with PBS, the sections were incubated at room temperature for 60 min with FOXM1 polyclonal rabbit-anti-human antibody (ProteinTech Group, China) at 1:100 dilution. After a PBS rinse, slides were then incubated for 25 min at room temperature with biotinylated goat-anti-rabbit immunoglobulin (Zhongshan Biotechnology, China) followed by incubation with peroxidase-conjugated streptavidin for 20 min and with fresh 0.05% 3, 3′-diaminobenzidine (DAB). Slides then were counterstained with Mayer’s hematoxylin and mounted on a crystal mount. As negative control for the staining procedure, the primary antibody was omitted. As positive controls epithelial ovarian cancer tissue that showed positive staining in earlier staining procedures was used. The percentage of cells positive for FOXM1 expression was graded and counted as follows: 1, 1-25%; 2, 26-50%; 3, 51-75%; and 4, 76-100%. Two pathologists who were blind of patients’ profiles observed the slides. The staining intensity score was graded as follows: 0 (no signal), 1 (weak), 2 (moderate), and 3 (marked). The scores for FOXM1 positivity and staining intensity were multiplied to obtain a final score, which determines FOXM1 expression as (- = 0; + = 1-4; ++ = 5-8; +++ = 9-12). In this study, we grouped all of the samples into the high expression group (++ or +++) and the low expression group (- or +) according to the protein expression.

### Cell lines and cell culture

ES-2, OVCAR-3, OVCA429, OVCA420 ovarian cancer cells were purchased from the American Type Culture Collection. HO-8910 and HO-8910 PM (a highly metastatic cell line derived from HO-8910) ovarian cancer cells were purchased from the Cell Bank of Type Culture Collection of the Chinese Academy of Sciences (Shanghai, China) [17]. All cell lines were incubated at 37°C under 5% CO_2_ in either minimum essential medium or Dulbecco’s modified Eagle medium with 10% fetal bovine serum and 1% Penicillin-Streptomycin.

### ShRNA and cDNA plasmid transfections

The coding regions of FOXM1 were inserted into pcDNA3.1 (Clontech). HO-8910 cells transfected with empty vectors (pcDNA3.1) were used as a control. HO-8910 PM cells were transfected with FOXM1 shRNA. A nonspecific control was used as non-targeting shRNAs. Transfections were performed with Lipofectamine 2000 reagent (Invitrogen, USA) using 1–2 mg of expression vector/ml serum-free medium as described by the manufacturer. The transfected cells were incubated for 24 h and harvested for real time PCR and western blot analysis.

### Gelatin zymography

MMP-2 and MMP-9 enzymatic activities in the cell culture medium were determined by SDS-PAGE gelatin zymography. After transfection with pcDNA3.1-FOXM1 or FOXM1 shRNA for 24 h, cells were continuously incubated in serum-free DMEM at 37°C for 24 h. The conditioned medium was then collected and centrifuged to remove cells and debris, and 30 μg of total protein was resolved in 7.5% polyacrylamide gels containing 0.1% gelatin. After electrophoresis, the enzymes were renatured by incubation with 2.5% Triton X-100 for 30 min at room temperature. The gels were then incubated in 50 mM Tris–HCl (pH = 7.5), containing 150 mM NaCl, 0.5 mM ZnCl_2_ and 10 mM CaCl_2_, for 24 h at 37°C. Gels were stained with coomassie blue and then destained with 10% acetic acid and 20% methanol in water. MMP-2 and MMP-9 activities were quantified by densitometry.

### RT-PCR

Total RNA was isolated from cultured cells with Trizol reagent (Invitrogen, USA). RT-PCR was performed using the Invitrogen SuperScript one-step RT-PCR kit according to manufacture’s instruction. The PCR conditions were as follows: 94°C for 2 min; 30 cycles of 94°C for 30 s, 56°C for 30 s, and 72°C for 60 s; and a final step of 10 min at 72°C. PCR products were analyzed using agarose gel electrophoresis and cloned into the pMD18-T (TaKaRa) vector for sequencing. The following primers were used: for FOXM1, 5′-GCGACAGGTTAAGGTTGAG-3′ (forward); 5′-AGGTTGTGGCGGATGGAGT-3′ (reverse); for β-actin, 5′-CGGGACCTGACTGACTACCTC-3′ (forward); 5′-TCGTCATACTCCTGCTTGCTG-3′ (reverse).

### Quantitative real-time PCR

Quantitative real-time PCR was carried out using real-time PCR with the SYBR Green reporter. The GAPDH was used as an internal control for each specific gene. Three independent experiments were performed to analyze the relative gene expression and each sample was tested in triplicate. The primers used for PCR were as follows: FOXM1 forward 5′-GCGACAGGTTAAGGTTGAG-3′; reverse 5′-AGGTTGTGGCGGATGGAGT-3′; MMP-2 forward 5′-TGATCTTGACCAGAATACCATCGA-3′; reverse 5′-GGCTTGCGAGGGAAGAAGTT-3′; MMP-9 forward 5′-CCTGGAGACCTGAGAACCAATC-3′; reverse 5′-CCACCCGAGTGTAACCATAGC-3′; VEGF-A forward 5′-CTTGCCTTGCTGCTCTACC-3′; reverse 5′-CACACAGGATGGCTTGAAG-3′; GAPDH forward 5′-GCACCGTCAAGGCTGAGAAC-3′; reverse 5′-TGGTGAAGACGCCAGTGGA-3′. Quantification was calculated using the comparative threshold cycle (Ct) method and efficiency of the RT reaction (relative quantity, 2^- ΔΔCt^).

### Western blot analysis

Cells were lysed in lysis buffer (phosphate-buffered saline containing 1% Triton X-100, protease inhibitor cocktail, and 1 mM phenylmethylsulfonyl fluoride) at 4°C for 30 min. Lysate protein concentration was estimated using BCA protein assay kit (Pierce, USA). Equal amounts of protein were electrophoresed under non-reducing conditions on 10% acrylamide gels, followed by transfer to a PVDF membrane on a semidry transfer apparatus. After being blocked with 5% nonfat milk for 1 h at room temperature, the membrane was incubated with antibodies against FOXM1, MMP-2, MMP-9, and VEGF-A (ProteinTech Group, China) with proper dilutions for 1 h, respectively. After washing, horseradish peroxidase-conjugated anti-rabbit IgG (Sigma, USA) was used as a secondary antibody and then incubated with the membrane for 1 h at room temperature. The immunoreactive proteins were then detected using the ECL system. Densitometric analysis of immunoblots was performed by using Quantity One software.

### Wound-healing assay

An in vitro wound-healing assay was used to assess cell motility. Ovarian cancer cells were plated at equal density in 24-well plates and grown to confluence. Wounds were then generated with a sterile pipette tip, cells were rinsed two times with PBS and fresh culture medium was added. Photos were taken at different time points under microscopy, and the wound healing was measured at 0 h, 12 h, 24 h, and 36 h. The experiment was done in triplicate.

### Cell migration and invasion assays

A Transwell system that incorporated a polycarbonate filter membrane with a pore size of 8 μm (BD Biosciences, USA) was used to assess cell migration and invasion [18]. For cell invasion assays, after 24 h transfection, cells were seeded on the upper chamber at a density of 3.0 × 10^5^ cells/well in serum-free medium. Medium containing 10% fetal bovine serum medium was applied to the lower chamber as chemoattractant. After 48 h incubation at 37°C, non-invasive cells remaining on the upper surface of the membrane were removed by wiping with cotton-tipped swabs. Cells which invaded through the matrix gel and were adherent to the lower surface of the filter were fixed with methanol, stained with 0.5% crystal violet, photographed, and counted. Cell migration assay was performed according to the protocol described above, except that the cells were added into the inserts with 24 h incubation without matrix gel pre-coated. Each test group was assayed in triplicate.

### Statistical analysis

Tests for association between immunohistochemical expression and clinicopathologic variables were computed using *χ*^2^-test or Fisher’s exact test. PFS and OS curves were calculated with the Kaplan-Meier method and were analyzed with the log-rank test. Univariate analysis and multivariable models were fit using a Cox proportional hazards regression model. The results are presented as means ± SD. The statistical significance of differences was determined by Student’s *t*-test in 2 groups and oneway ANOVA in multiple groups. *P* < 0.05 was considered statistically significant. All data were analyzed with SPSS 13.0 software.

## Results

### Clinical significance of FOXM1 expression in EOC

**Figure 1:**
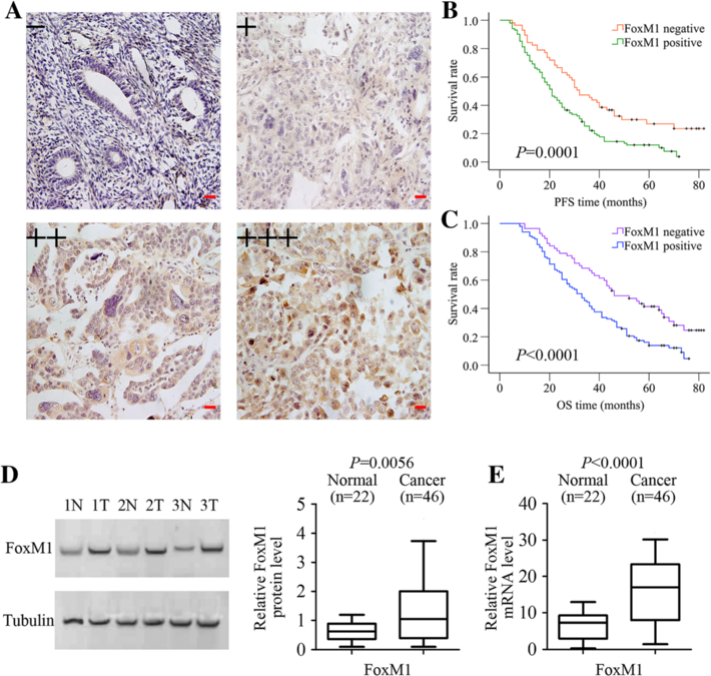
**FOXM1 expression level and its prognostic effects in epithelial ovarian cancer. A** Representative immunohistochemical stainings of FOXM1 in ovarian cancer (-, +, ++, +++). Scale bars = 0.1 mm. **B** Kaplan-Meier progression-free survival curves for FOXM1-negative patients with EOC (n = 57) and FOXM1-positive patients with EOC (n = 101). **C** Kaplan-Meier overall survival curves for FOXM1-negative patients with EOC (n = 57) and FOXM1-positive patients with EOC (n =101). **D** The FOXM1 protein expression was higher in 46 EOC tissues than in 22 normal ovarian tissues (*P* = 0.0056). **E** The relative mRNA expression of FOXM1 was higher in 46 EOC tissues than in 22 normal ovarian tissues (*P* < 0.0001).

As shown in Figure 1A, FOXM1 was mainly expressed in the cytoplasm and nucleus of EOC cells using immunostaining. In the majority of cases, ovarian cancer cells showed both a cytoplasmic and nuclear staining for FOXM1, but staining was restricted to cytoplasm or nuclear in a few cases. By definition, we grouped all of the samples into the high expression group (both a cytoplasmic and nuclear staining for FOXM1) and the low expression group according to the protein expression. The correlation between FOXM1 expression and clinicopathological parameters such as age, FIGO stage, grade, histological type and lymph node status was summarized in Table 1. The expression of FOXM1 in patients with lymph node metastasis was significantly higher than in patients without lymph node metastasis (*P* = 0.009). The Kaplan-Meier survival analysis showed that FOXM1 expression had a significant adverse effect on survival (PFS, *P* = 0.0001, Figure 1B; OS, *P* < 0.0001, Figure 1C). By using univariate Cox proportional analysis, FOXM1 expression was statistically correlated to survival (PFS, *P* = 0.001, Table 2; OS, *P* = 0.001, Table 3). Multivariate Cox analysis showed that FIGO stage, lymph node metastasis and FOXM1 expression were independent risk factors that influenced the prognosis of EOC (Table 2 and 3).

**Table 1:** Relationship between FOXM1 protein expression with clinicopathological features of patients

**Characteristic**	**No.**	**FOXM1 protein expression**		***P***
		**Negative**	**Positive**	
Age				0.922
<60	99	36	63	
≥60	59	21	38	
FIGO stage				0.127
I + II	44	20	24	
III + IV	114	37	77	
Grade				0.298
G1	24	12	12	
G2	46	16	30	
G3	88	29	59	
Histological type				0.481
Serous	69	27	42	
Nonserous	89	30	59	
Lymph node metastasis				0.009*
Negative	120	50	70	
Positive	38	7	31	

*P < 0.05.

**Table 2:** Univariate and multivariate analyses of PFS in patients with epithelial ovarian cancer

**Variables**	**Categories**	**Univariate analysis**		***P***	**Multivariate analysis**		***P***
		**HR**^**a**^	**95% CI**^**b**^		**HR**^**a**^	**95% CI**^**b**^	
Age	<60						
	≥60	1.061	0.744-1.512	0.744	—	—	—
FIGO stage	I + II						
	III + IV	2.081	1.371-3.159	0.001*	1.789	1.170-2.733	0.007*
Grade	G1						
	G2	0.909	0.523-1.581	0.735	—	—	—
	G3	1.291	0.789-2.113	0.309	—	—	—
Histological type	Serous						
	Nonserous	1.059	0.750-1.496	0.744	—	—	—
Lymph node metastasis	Negative						
	Positive	2.988	2.028-4.403	<0.001*	2.308	1.531-3.480	<0.001*
FOXM1	Negative						
	Positive	1.878	1.292-2.729	0.001*	1.494	1.007-2.215	0.046*

*PFS* progression-free survival, ^a^*HR* = hazard ratio, ^b^*CI* = confidence interval, *P < 0.05.

**Table 3:** Univariate and multivariate analyses of OS in patients with epithelial ovarian cancer

**Variables**	**Categories**	**Univariate analysis**		***P***	**Multivariate analysis**		***P***
		**HR**^**a**^	**95% CI**^**b**^		**HR**^**a**^	**95% CI**^**b**^	
Age	<60						
	≥60	1.236	0.868-1.761	0.239	—	—	—
FIGO stage	I + II						
	III + IV	2.263	1.467-3.490	<0.001*	1.996	1.285-3.099	0.002*
Grade	G1						
	G2	1.056	0.608-1.833	0.847	—	—	—
	G3	1.234	0.744-2.049	0.416	—	—	—
Histological type	Serous						
	Nonserous	1.108	0.780-1.573	0.568	—	—	—
Lymph node metastasis	Negative						
	Positive	2.856	1.922-4.243	<0.001*	2.142	1.411-3.251	<0.001*
FOXM1	Negative						
	Positive	1.960	1.338-2.871	0.001*	1.599	1.069-2.391	0.022*

*OS* overall survival, ^a^*HR* = hazard ratio, ^b^*CI* = confidence interval, *P < 0.05.

### Expression levels of FOXM1 in ovarian cancer cells and ovarian tissues

**Figure 2:**
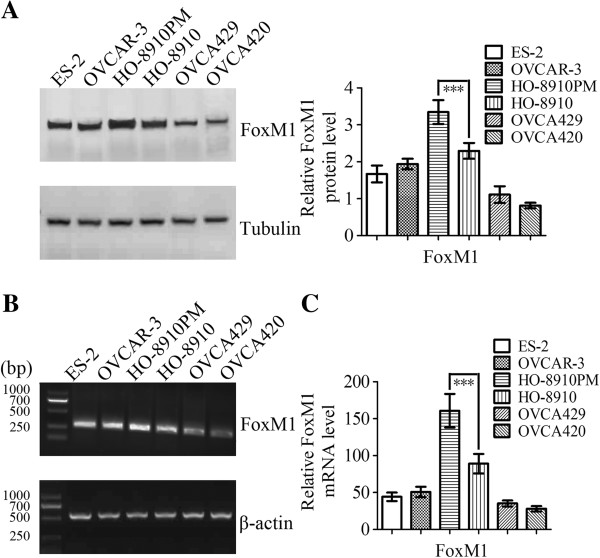
**Expression levels of FOXM1 in ovarian cancer cells. A** Expression levels of FOXM1 proteins in ovarian cancer cells detected by western blot. Levels of tubulin were evaluated as an internal control for loading. **B** RT-PCR analysis of FOXM1 mRNA expression levels in ovarian cancer cells. **Β**-actin was amplified as a control. **C** Expression levels of FOXM1 mRNA in ovarian cancer cells detected by real-time PCR. The mRNA expression levels were normalized against GAPDH. Columns mean derived from at least three independent experiments, bars, SD. Statistical analysis was done using Student’s t tests. ***P < 0.001, significant.

To assess the FOXM1 expression levels, we compared the levels of FOXM1 expression in normal ovarian tissues, ovarian cancer tissues and ovarian cancer cell lines by real-time PCR and western blot analyses. Expression levels of FOXM1 mRNA and protein were shown to be significantly increased in ovarian cancer tissues compared with normal ovarian tissues (Figure 1D and 1E). We further detected the expression of FOXM1 in ovarian cancer cells. FOXM1 was detectable in all these six cell lines, with highest expression in HO-8910 PM cells. As shown in Figure 2A and 2C, expression of FOXM1 protein and mRNA in HO-8910 cell line was lower than that in its subline cell line HO-8910 PM (a highly metastatic cell line derived from HO-8910, *P* < 0.001). We then detected the expression of FOXM1 in these same cells using RT-PCR assay (Figure 2B).

### MMP-2, MMP-9 and VEGF-A expression after transfection with FOXM1

**Figure 3:**
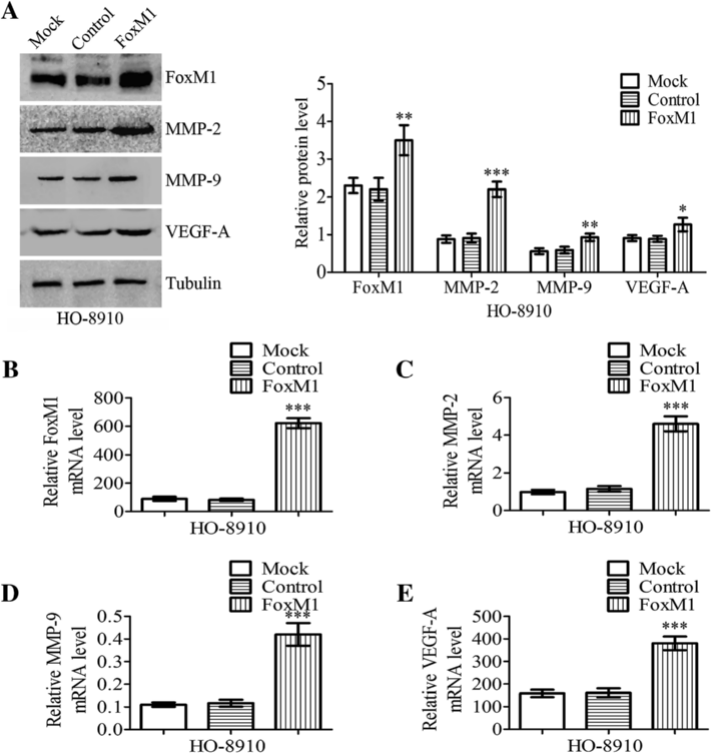
**Effects on MMP-2, MMP-9 and VEGF-A expressions of HO-8910 cells after transfection with FOXM1.** HO-8910 cells were either untransfected (Mock), or transfected with pcDNA3.1 (control) or pcDNA3.1-FOXM1 (FOXM1). **A** Western blot demonstrated FOXM1, MMP-2, MMP-9 and VEGF-A protein levels were significantly increased after transfection with FOXM1. The tubulin was analyzed as a control. **B**-**E** FOXM1, MMP-2, MMP-9 and VEGF-A mRNA expression levels of HO-8910 cells after transfection with FOXM1 were quantified by real-time PCR using GAPDH as a reference. Statistical significance was assessed using one-way ANOVA. Data are presented as mean ± SD for three independent experiments. *P < 0.05, **P < 0.01, ***P < 0.001, statistically significant difference.

**Figure 4:**
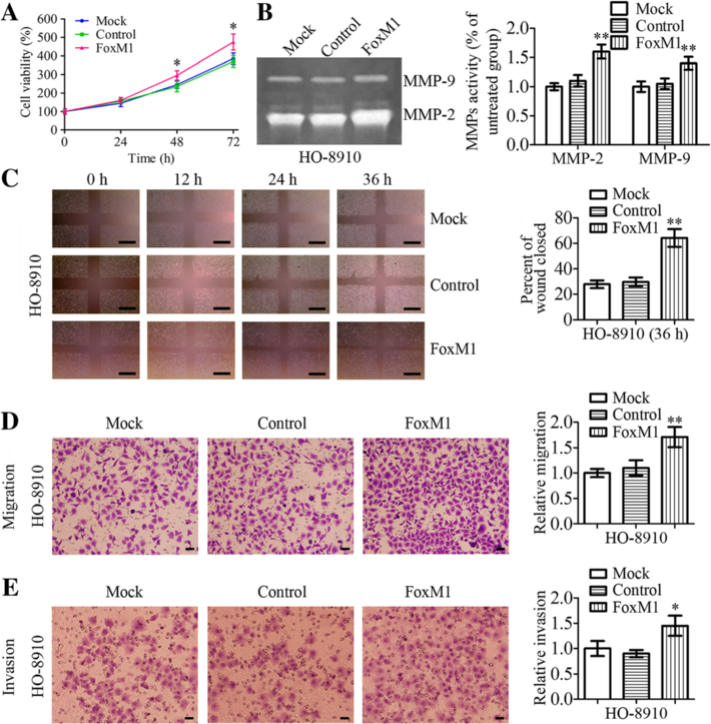
**Effects on cell proliferation, migration, invasion and MMP-9/MMP-2 activities of HO-8910 cells after transfection with FOXM1.** HO-8910 cells were either untransfected (Mock), or transfected with pcDNA3.1 (control) or pcDNA3.1-FOXM1 (FOXM1). **A** Cell proliferation rates were determined by MTT assay. **B** HO-8910 cells were incubated with serum-free media for 24 h and conditioned media were collected and subjected to gelatin zymography. Zymographic analysis showed increased MMP-2 and MMP-9 activity in FOXM1-transfected HO-8910 cells. **C** Wound healing assays were done to determine the effects on cell migration of HO-8910 cells after transfection with FOXM1. The wound gaps were measured at six reference points along the wound, and the results were expressed as the average wound gap. Scale bars = 0.5 mm. **D**-**E** HO-8910 cells were transfected with mock, control or FOXM1 and then subject to transwell migration/invasion assays, as described in Methods. After 24/48 h, migratory/invasive cells were counted after staining with crystal violet. Scale bars = 0.1 mm. Statistical significance was assessed using one-way ANOVA. Data are presented as mean ± SD for three independent experiments. *P < 0.05, **P < 0.01, statistically significant difference.

HO-8910 cells were either untransfected (Mock), or transfected with pcDNA3.1 (control) or pcDNA3.1-FOXM1 (FOXM1), as shown in Figure 3 and Figure 4. After transfection of cells, real-time PCR and western blot analyses were performed to analyze the expression levels of FOXM1 mRNA and protein. As shown in Figure 3A and 3B, the expression of FOXM1 mRNA and protein was significantly increased in pcDNA3.1-FOXM1-transfected cells compared with pcDNA3.1-transfected cells and untransfected cells (*P* < 0.001, *P* < 0.01, respectively). Moreover, we investigated the protein expression and mRNA expression of MMP-2, MMP-9 and VEGF-A by real-time PCR and western blot analyses. As shown in Figure 3A, the expression of MMP-2, MMP-9 and VEGF-A protein in pcDNA3.1-FOXM1-transfected cells was increased compared to those in pcDNA3.1-transfected cells and untransfected cells (*P* < 0.001, *P* < 0.01, *P* < 0.05, respectively). Similar results were observed by real-time quantitative PCR analysis as well, as shown in Figure 3C, 3D and 3E (*P* < 0.001). To investigate the effects of FOXM1 transfection on MMP-2 and MMP-9 enzyme activities, gelatin zymography were performed to examine the activities of MMP-2 and MMP-9 using conditioned medium, which was collected and measured after 24 h transfection. The results showed that MMP-2 and MMP-9 enzymatic activities were significantly increased in pcDNA3.1-FOXM1-transfected cells, compared to those in pcDNA3.1-transfected cells and untransfected cells (Figure 4B, *P* < 0.01).

### Effects of FOXM1 overexpression on cell proliferation, migration and invasion

To confirm the effects of FOXM1 overexpression on cell proliferation, FOXM1 was overexpressed in HO-8910 cells after transfection with pcDNA3.1-FOXM1. The cells were assessed for cell proliferation by MTT assay. Our results revealed that pcDNA3.1-FOXM1-transfected cells showed a significantly higher proliferation rate than pcDNA3.1-transfected cells and untransfected cells (Figure 4A).

Next, we determined the effects of FOXM1 overexpression on tumor cell metastasis. Cellular migration was analysed by wound-healing assay while invasiveness was analysed with transwell experiments. The results of wound-healing assay and transwell migration assay demonstrated that HO-8910 cells that were transfected with the pcDNA3.1- FOXM1 plasmid exhibited a significant increase in cellular migration as compared with control cells (Figure 4C and 4D, *P* < 0.01). In the *in vitro* invasion assays, the number of cells invaded through the transwell membrane in pcDNA3.1-FOXM1-transfected group was significantly higher than those in the control group (Figure 4E, *P* < 0.05).

### MMP-2, MMP-9 and VEGF-A expression after transfection with FOXM1 shRNA

**Figure 5:**
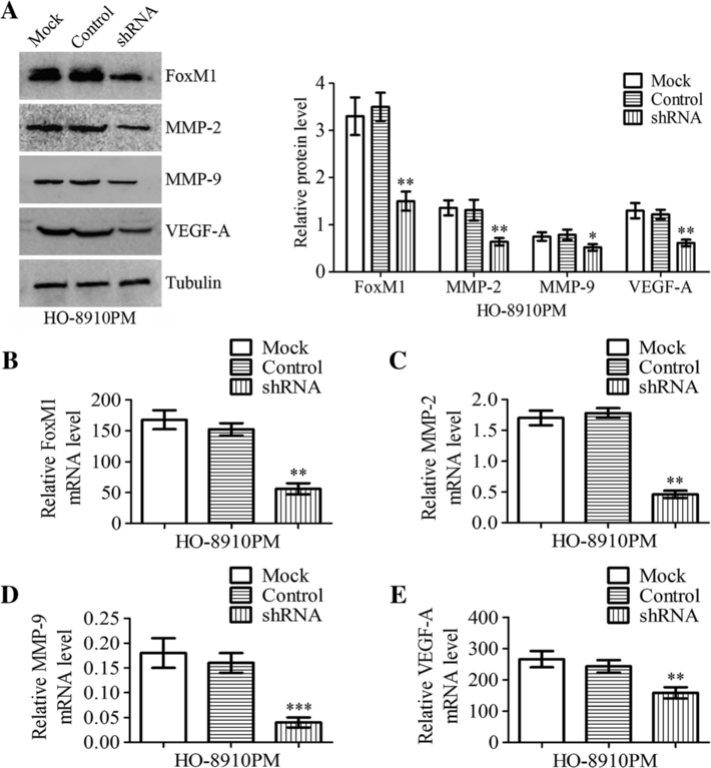
**Effects on MMP-2, MMP-9 and VEGF-A expressions of HO-8910 PM cells after transfection with FOXM1 shRNA.** HO-8910 PM cells were either untransfected (Mock), or transfected with nonspecific shRNA (control) or FOXM1 shRNA (shRNA). **A** Western blot demonstrated FOXM1, MMP-2, MMP-9 and VEGF-A protein levels were significantly reduced after transfection with FOXM1 shRNA. The tubulin was analyzed as a control. **B**-**E** FOXM1, MMP-2, MMP-9 and VEGF-A mRNA expression levels of HO-8910 PM cells after transfection with FOXM1 shRNA were quantified by real-time PCR using GAPDH as a reference. Statistical significance was assessed using one-way ANOVA. Data are presented as mean ± SD for three independent experiments. *P < 0.05, **P < 0.01, ***P < 0.001, statistically significant difference.

**Figure 6:**
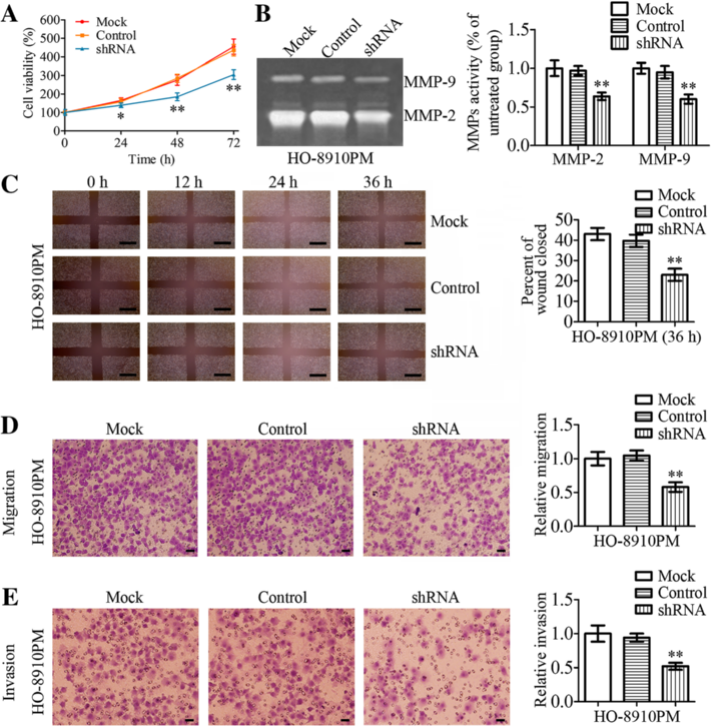
**Effects on cell proliferation, migration, invasion and MMP-9/MMP-2 activities of HO-8910 PM cells after transfection with FOXM1 shRNA.** HO-8910 PM cells were either untransfected (Mock), or transfected with nonspecific shRNA (control) or FOXM1 shRNA (shRNA). **A** Cell proliferation rates were determined by MTT assay. **B** HO-8910 PM cells were incubated with serum-free media for 24 h and conditioned media were collected and subjected to gelatin zymography. Zymographic analysis showed decreased MMP-2 and MMP-9 activity in HO-8910 PM cells transfected with FOXM1 shRNA. **C** Wound healing assays were done to determine the effects on cell migration of HO-8910 PM cells after transfection with FOXM1 shRNA. The wound gaps were measured at six reference points along the wound, and the results were expressed as the average wound gap. Scale bars = 0.5 mm. **D**-**E** HO-8910 PM cells were transfected with mock, control or FOXM1 shRNA and then subject to transwell migration/invasion assays, as described in Methods. After 24/48 h, migratory/invasive cells were counted after staining with crystal violet. Scale bars = 0.1 mm. Statistical significance was assessed using one-way ANOVA. Data are presented as mean ± SD for three independent experiments. *P < 0.05, **P < 0.01, statistically significant difference.

HO-8910 PM cells were either untransfected (Mock), or transfected with nonspecific shRNA (control) or FOXM1 shRNA (shRNA), as shown in Figure 5 and Figure 6. After transfection for 24 h, real-time PCR and western blot analyses were performed to analyze the expression levels of FOXM1 mRNA and protein. As shown in Figure 5A and 5B, the expression of FOXM1 mRNA and protein was significantly decreased in FOXM1 shRNA-transfected cells compared with control shRNA-transfected cells and untransfected cells (*P* < 0.01). Moreover, we investigated the protein expression and mRNA expression of MMP-2, MMP-9 and VEGF-A by real-time PCR and western blot analyses. As shown in Figure 5A, the expression of MMP-2, MMP-9 and VEGF-A protein in FOXM1 shRNA-transfected cells was decreased compared to those in control shRNA-transfected cells and untransfected cells (*P* < 0.01, *P* < 0.05, *P* < 0.01, respectively). Similar results were observed by real-time quantitative PCR analysis as well, as shown in Figure 5C, 5D and 5E (*P* < 0.01, *P* < 0.001, *P* < 0.01, respectively). To investigate the effects of FOXM1 shRNA transfection on MMP-2 and MMP-9 enzyme activities, gelatin zymography were performed to examine the activities of MMP-2 and MMP-9 using conditioned medium, which was collected and measured 24 h after transfection. The results showed that MMP-2 and MMP-9 enzymatic activities were significantly reduced in FOXM1 shRNA-transfected cells, compared to those in control shRNA-transfected cells and untransfected cells (Figure 6B, *P* < 0.01).

### Effects of FOXM1 silencing on cell proliferation, migration and invasion

To confirm the effects of FOXM1 silencing on cell proliferation, FOXM1 was down-regulated in HO-8910 PM cells using shRNA against FOXM1 transcripts. The cells were assessed for cell proliferation by MTT assay. Our results revealed that FOXM1 shRNA-transfected cells showed a significantly lower proliferation rate than control shRNA-transfected cells and untransfected cells (Figure 6A).

Next, we determined the effects of FOXM1 silencing on tumor cell metastasis. Cellular invasiveness and migration were analyzed by wound-healing assay and transwell assays, respectively. The results of wound-healing assay and transwell migration assay demonstrated a significant reduction in motility of FOXM1 shRNA-transfected cells compared with the control groups (Figure 6C and 6D, *P* < 0.01). In the *in vitro* invasion assays, the number of cells invaded through the transwell membrane in FOXM1 shRNA-transfected group was significantly lower than those in the control group (Figure 6E, *P* < 0.01).

## Discussion

Previous studies have shown that the expression of FOXM1 was increased in different types of cancer. Importantly, some studies also have evaluated the prognostic significance of FOXM1 in several types of cancer [12-16]. However, the clinical significance of FOXM1 expression in EOC had not been determined prior to this study. This is the first study determining the influence of FOXM1 expression on the prognosis of EOC. In this study of 158 patients with EOC with long-term follow-up available, we analyzed the prognostic significance of FOXM1 expression in terms of survival and its association with clinicopathologic variables. FOXM1 protein expression was examined immunohistochemically in EOC, and FOXM1 was mainly located in the nucleus and cytoplasm. A statistically significant relationship was found for FOXM1 expression with lymph node metastasis and FOXM1 expression was significantly associated with patients’ survival (PFS, *P* = 0.0001, Figure 1B; OS, *P* < 0.0001, Figure 1C). Several studies have now been published that FOXM1 expression correlates with tumor stage, grade and may have prognostic utility [19-23]. Correlations between FOXM1 and clinicopathologic parameters have been reported in ovarian cancer, but the results were inconsistent [20]. Our results indicated FOXM1 expression to be significantly associated with lymph node metastasis (*P* = 0.009), but not with FIGO stage (*P* = 0.127) and grade (*P* = 0.298). The discrepancy of immunohistochemical results may be explained partly by the different antibody used, because they used monoclonal antibody. Another limitation of our study was the relatively small population of patients that we evaluated. In multivariate analysis, FOXM1 expression was identified as an independent prognostic factor for both PFS and OS (Table 2 and 3). We also found that FOXM1 was highly expressed in most primary EOC tissues but lowly expressed in normal ovarian tissues at both the protein (*P* = 0.0056, Figure 1D) and the mRNA level (*P* < 0.0001, Figure 1E). These data suggests that FOXM1 may not only be involved in the progression of EOC, but also in the metastasis, and therefore might be a reasonable target for directed therapeutics.

We validated the correlation between FOXM1 expression and tumor progression and metastasis by *in vitro* functional studies. The following study began with the use of real-time PCR and western blot to identify genes differentially expressed in two clonally related human EOC cell lines differing in metastatic activity, and this revealed a significant difference in FOXM1 expression. The results showed that FOXM1 protein and mRNA were lowly expressed in HO-8910 but were highly expressed in its more metastatic derivative, HO-8910 PM (Figure 2A and 2C) [17]. Diagnosis of epithelial ovarian cancer usually occurs when the cancer has already progressed to the advanced stages [2]. Metastasis remains the major problem in managing EOC, and invasion is the first step of metastasis. Thus, blocking the invasion and metastasis of cancer cells is of great significance in EOC treatment.

To test the significance of FOXM1 interference in EOC cells, we transfected pcDNA3.1-FOXM1 plasmid and FOXM1 shRNA into HO-8910 cells and HO-8910 PM cells, respectively. Cell growth, migration and invasion are important processes involved in tumor progression. In our study, we explored whether FOXM1 contributed to cell growth, migration and invasion of EOC cells in vitro. The results showed that overexpression of FOXM1 by transfection with pcDNA3.1-FOXM1 could promote cell growth, invasion and metastasis. Similarly, we found that depletion of FOXM1 by transfection with FOXM1 shRNA could suppress cell growth, invasion and metastasis. Several studies have shown that FOXM1 could promote cell growth, invasion and metastasis in various cell types [4,5,24,25]. Here, we reached the same conclusion in EOC. To our knowledge, this study is novel in investigating the role and mechanisms of FOXM1 in invasion and metastasis of EOC cells.

The present study suggested that FOXM1 expression was closely associated with increased tumor invasion, migration and metastasis. It has been reported that a number of FOXM1 downstream target molecules are involved in regulating tumor progression and invasive behaviors. In all these processes, MMP-2, MMP-9 and VEGF-A are thought to play a critical role in EOC cells. Among matrix metalloproteases (MMPs), a family of zinc dependent endopeptidases, MMP-2 and MMP-9 have been considered to be critical for tumor growth, invasion and metastasis [26,27]. It is also known that VEGF-A is another important molecule that is involved in tumor growth, invasion and metastasis [28,29]. Moreover, some studies have documented that overexpression of MMP-2, MMP-9 and VEGF-A was associated with cancer progression and metastasis in ovarian cancer [30-32]. Our *in vitro* data indicated that the expressions of MMP-2, MMP-9 and VEGF-A were obviously increased in pcDNA3.1-FOXM1-transfected HO-8910 cells, however they were obviously decreased in FOXM1 shRNA-transfected HO-8910 PM cells. Previous research has demonstrated that up-regulation of FOXM1 increased the expression of MMP-2, MMP-9 and VEGF-A, resulting in the promotion of proliferation, migration and invasion of cancer cells [9,15,33]. Our results emphasize the conclusion that FOXM1 regulates the expression of MMP-2, MMP-9 and VEGF-A in EOC cells. These results suggest that downregulation of FOXM1 could potentiate antimetastatic activity partly through down-regulating expressions of MMP-2, MMP-9 and VEGF-A in EOC. However, it is not clearly understood how FOXM1 regulates the expression of MMP-2, MMP-9 and VEGF-A in EOC cells. Further studies are required to distinguish the possible interaction between FOXM1 and the above proteins.

## Conclusions

In summary, the present study showed that FOXM1 overexpression was associated with lymph node status and poor patient survival in EOC. Our study demonstrated that FOXM1 played an important role in proliferation, migration and invasion of EOC. Moreover, we demonstrated that FOXM1 regulated the expression of MMP-2, MMP-9 and VEGF-A in EOC cells. Taken together, our results suggest that elevated FOXM1 may be a prognostic marker of EOC and that FOXM1 may serve as a promising therapeutic target for inhibition of ovarian cancer progression.

## Abbreviations

EOC: Epithelial ovarian cancer; MMP-2: Matrix metalloproteinase-2; MMP-9: Matrix metalloproteinase-9; VEGF-A: Vascular endothelial growth factor-A; PFS: Progression-free survival; OS: Overall survival; FIGO: International Federation of Gynecology and Obstetrics.

## Competing interests

The authors declare that they have no competing interests.

## Authors’ contributions

All authors read and approved the final manuscript. NW, HY, WCL and YL are responsible for the study design. NW, YuW, SHZ and LW performed the experiments and draft the manuscript. HXL, SHZ and ZHA collected the data. YuW, BW, YiW, and LW participated in the data analysis and interpretation.
